# Prevalence of Psychiatric Disorders among the Rural Geriatric Population: A Pilot Study in Karnataka, India

**DOI:** 10.5195/cajgh.2015.138

**Published:** 2015-03-10

**Authors:** Sreejith S. Nair, Pooja Raghunath, Sreekanth S. Nair

**Affiliations:** 1 Department of Community Medicine, Navodaya Medical College, Raichur, Karnataka, India;; 2 Department of Microbiology, Pushpagiri Institute of Medical Sciences and Research Center, Tiruvalla, Kottayam, Kerala, India;; 3 Department of Forensic Medicine, Academy of Medical Sciences, Pariyaram, Kannur, Kerala, India

**Keywords:** psychiatric disorder, depression, anxiety, geriatric, aging, India

## Abstract

**Background:**

Increasing life expectancy around the world, an outstanding achievement of our century, has brought with it new public health challenges. India is the second most populous country in the world, with over 72 million inhabitants above 60 years of age as of 2001. The life expectancy in India increased from 32 years in 1947 to over 66 years in 2010, with 8.0% of the population now reaching over 60 years of age. Few studies in India target the health, especially mental health, of this geriatric population. This study aims to estimate the current prevalence of psychiatric disorders in the geriatric population of the rural area of Singanodi,Karnataka, India.

**Methods:**

This cross sectional, epidemiological, community-based study was conducted in a rural health training area of Singanodi, Raichur District, Karnataka, India.The General Health Questionnaire-12, Mini Mental State Examination, and Geriatric Depression Scale were administered to 366 participants. Chi square tests with Yates correction were utilized for statistical analysis using SPSS 19.0 software.

**Results:**

We found that 33.9% of the geriatric population in the selected province were above the threshold for mental illness based on the GHQ-12 questionnaire. Females had a higher prevalence of mental disorder at 77.6% (152 out of 196) as compared to males who had a prevalence of 42.4% (72 out of 170). The most common psychiatric disorder was depression (21.9%), and generalized anxiety was present in 10.7% of the study population. Prevalence of cognitive impairment was 16.3%, with a significantly higher percentage of affected individuals in 80+ age group.

**Conclusion:**

Mental disorders are common among elderly people, but they are not well documented in rural India. The assessment of psychiatric disorder prevalence will help strengthen psycho-geriatric services and thus improve the quality of life of the elderly. A system that ensures comprehensive health care will have to be developed for this purpose as part of our future efforts.

Aging refers to the multidimensional process of physical, psychological, and social change.^1^ Recent advances in health sciences and improvement in social conditions have led to an increase in life expectancy in most countries of the world.^2^ However, increased life expectancy around the world also brought new public health challenges, such as increasing incidence and prevalence of chronic, age-related disorders.^3^

In India, the second most populous country in the world, the proportion of those 60 years and older was 5.4% in 1951, and it increased to 8.0% in 2010.^4^ Life expectancy at birth for males increased from 42 years (1951–1960) to 58 years (1986–1990).^4^ Life expectancy is projected to increase to 67 years for males and 69 years for females by the year 2016.^5^ Furthermore, the United Nations indicated that 21.0% of the Indian population will be aged 60+ years by 2050.^5^

Mental disorders in the elderly often go untreated due to the misperceptions that these disorders are a normal part of aging and a natural reaction to chronic illness, loss of family members, and social transition occurring with age.^6^ The burden of late-life psychiatric disorder on physical health, social support systems, and overall functioning is considerable, making mental disorders a leading cause of burden in elderly adults.^7^ Additionally, mental disorder is a preventable risk factor for mortality, particularly suicide attempts.^8^

Western countries have conducted numerous studies on the resources, needs, and outcomes on the community-based care of the elderly, which helped in the estimation of public health burden of the geropsychiatric population.^9^–^14^ Few studies have been conducted in India on the extent of mental disorder burden in these geriatric age groups. Pathak^14^ noted that there have been few publications on the health problems of those aged 60 years and above in India,^15^ while even fewer have examined the mental health of the elderly in India.^16^ The purpose of this article is to highlight the psychiatric problems faced by the elderly Indian population as well as develop strategies to improve the quality of life for the elderly.^17^

## Methods

This cross-sectional, observational, community-based study was conducted in the rural health training area of Singanodi, Raichur District, Karnataka, India.The Navodaya Medical College and Research Centre Institutional Ethical Review Board approval was obtained before commencing the study. Informed consent was obtained prior to study participation.

### Study Population

We used the United Nations (UN) guideline of 60+ years to refer to the elderly population.^18^ The area of Singanodi has a population of 25,486 with a geriatric population of approximately 2,500 residents. A sample size of 383 was estimated using the formula 4pq/L^2^ (prevalence of 42%,^19^ allowable error 12% and 95% confidence).

Of the 383 elderly participants, 17 persons could not be included in the study due to the individuals or their family members’ refusal to participate. Thus, a total of 366 were included in the final sample.

### Procedures

The team made twenty visits between January 15 and April 15, 2014. A community medicine postgraduate physician and 3 social workers visited the study area once or twice a week. Prior to the start of the study, the team members underwent training in the use of the screening devices and a degree of standardization was achieved. All interviewers were trained in the standard operating procedure of survey administration to avoid any information bias.

Commencing from the eastern end of the town, a door-to-door survey was implemented. Residents of the houses were querried for the presence of any resident aged 60 years and above in the house. If due to some reason the potential participant was not available during first visit, he/she was contacted during the subsequent visit. Inclusion criteria for the study were: aged 60 years or above at the time of survey, a resident of the study area (Singanodi) for at least one year prior to the start of the study or those staying for less than a year but intended to stay permanently. Individuals were excluded if they were guests or lived in the area for less than one year and did not intend to stay permanently.

### Measures

Four survey instruments were utilized. First, the General Health Questionnaire-12 (GHQ-12)^20^,^21^ is a self-administered screening test, which is the most commonly used screening instrument for detecting psychiatric disorders in community settings and non-psychiatric clinical settings. A score of ≥2 is the cut-off score for possible psychiatric disorder for this screening instrument.^20^ Second, the Mini-Mental State Examination (MMSE) is the most widely used cognitive screening instrument worldwide.^22^ It is commonly used to screen for dementia. Any score ≥27 points indicates a normal cognition. Below this, scores can indicate severe (<9 points), moderate (10–18 points) or mild (19–26 points) cognitive impairment. The Hindi translation of MMSE that was suitably modified was used in this study, which has been validated in various studies.^16^–^18^ Third, the Geriatric Depression Scale-15, short version (GDS) is a 15 item self-report scale for assessing depression.^23^ In this scale, scores of 0–9 are considered normal, 10–19 indicated mild depression, and 20–30 indicated severe depression. Fourth, the Generalized Anxiety (GA) Scale was adapted from the CARE^24^ schedule as a subscale. Scores of 5–9 points is indicative of mild anxiety, and a score of 10 points or higher is indicative of major anxiety.^19^–^21^

### Data Analysis

Descriptive statistics were used to gather basic participant characteristics as well as the prevalence of psychiatric disorders. Chi square analyses with a Yates correction were used to analyze age group differences for psychiatric disorder prevalence and to analyze gender differences for psychiatric disorder prevalence. All analyses were conducted using SPSS 19.0 software.

## Results

There were 366 persons from 205 households aged 60 years and above in the surveyed population who agreed to participate in the study, with women comprising 53.6% of the sample. The distribution of participants in each age group was similar for both the sexes. Table 1 shows the distribution of the sample population according to age and gender.

The majority of participants were in 60–64 age group (42%). Table 2 shows the distribution of psychiatric disorder prevalence stratified by age, sex, and marital status.

Presence of psychiatric morbidity was defined as having screened positive for at least one of the following: cognitive decline, dementia, depression, or generalized anxiety. Participants in the age group 80+ screened positive for more psychiatric disorders as compared to younger age groups (X^2^ = 10.25, *p* < 0.05). Similarly, significantly more females were mentally ill as compared to males (X^2^ = 23.75, *p* < 0.001). Further, we observed that significantly more widowed participants have been affected by mental disorders compared to married participants (X^2^ = 25.17, *p* < 0.001).

### Prevalence of psychiatric disorders

33.9% had scores ≥2 in GHQ-12, i.e. above the cut-off score for possible psychiatric disorder for this screening instrument and requiring further mental health evaluation (Table 3).

Of these subjects, cognitive impairment was present in 60 participants (16.3%). Depression was present in 80 (21.9%) of the study participants. Generalized anxiety was present in 39 (10.66%) study participants. There was a significant effect of age on having a diagnosable disorder based on the GHQ-12 (*p*=0.03) and cognitive impairment based on he MMSE (*p*=0.03). However, there was no statistically significant effect of age on depression (*p*=0.82) and anxiety (*p*=0.87).

## Discussion

This study demonstrated that the prevalence of mental disorder was 33.9% of the elderly population (60 years and older). One previous study estimated that he prevalence of mental disorder in those 50 years and older was 34.9% in the area of Madras, India.^25^ Another study conducted in the UK estimated that the prevalence of mental disorder in those 65 years and older was 46.0%.^13^,^26^ In our sample, the burden of mental disorder was higher in females, corroborating the findings of many studies conducted in India^25^,^27^–^29^ and western countries.^30^,^31^

The most prevalent disorder amongst the elderly population, as reported by many field-surveys conducted in India and abroad, was depression. Depression was found in 16.4% of the population, which is similar to a 13.3–18.3% prevalence reported in the literature.^32^,^33^ The prevalence rates in Indian studies have been widely varied, ranging from 6.0%to 55.2%.^34^ Banerjee and MacDonald^13^ found that depression was prevalent in 26.0% of their sample comprising persons aged 65 years and above. A significant finding of this study, which may have important implications for both social and psychological perspectives, is the high prevalence of psychiatric disorder amongst widowed people. Stressful factors such as isolation and low socioeconomic status are closely associated with widowhood.

In the present study, 10.66 % of the persons had GAD, which is similar to the 4.6% prevalence rate reported by Ritchie et al.^35^ Most Indian researchers reported a low prevalence of anxiety disorders in the elderly population.^25^,^34^

It is therefore evident that the mental health care needs of the elderly are multifaceted. A system that ensures a comprehensive health care needs to be developed for this purpose. We should not, however, lose sight of the fact that provision of health facilities does not necessarily ensure its adequate utilization.^36^,^37^

### Strengths and Limitations

One limitation of this study is that all of the study participants were from one rural location instead of multiple sites. Future studies wanting to understand the impact of psychological disorders among the elderly in rural populations could focus on a multi-centric approach, using cohorts from multiple rural populations. Another limitation is that the data was gathered by self-report methods, which might cause bias due to the fact that the study population is relatively small. Major strengths of this study are the inclusion of reliable screening questionnaires and standardization of interviewers to reduce bias.

## Conclusion

There are many barriers to the utilization of health facilities by the community, with more barriers experienced by the elderly. Apart from their limited mobility, limited information access, and inadequate awareness of treatability of mental disorders, the elderly are likely to experience a lack of family support and social isolation. The basic philosophy of geriatric research is neither the prevention of old age nor a mere addition of years, but to “add life to years.” By assessing the social and familial risk factors of mental disorder among elderly persons residing in a rural community, community-based rehabilitation and suicide prevention programs could be developed. Raising awareness about mental disorders and its association with the geriatric age group may be an effective measure for the early detection and treatment of such disorders.

## Figures and Tables

**Table 1:** Distribution of study population age and sex

**Age Group**	*N* (%)	

	Male	Female
60 – 64	80 (47.0)	74 (37.7)
65 – 69	52 (30.6)	58 (29.6)
70 – 74	16 (9.4)	28 (14.3)
75 – 79	10 (5.9)	18 (9.2)
80+	12 (7.1)	18 (9.2)
Total	170 (100.0)	196 (100.0)

**Table 2:** Distribution of psychiatric disorder prevalence stratified by age, sex, and marital status

Variable	N (%)	

	Normal Screen	Positive Screen
Age Group*		
60 – 64	62 (44.9)	92 (40.4)
65 – 69	50 (36.2)	60 (26.3)
70 – 74	8 (5.8)	36 (15.8)
75 – 79	14 (10.2)	14 (6.1)
80+	4 (2.9)	26 (11.4)
Gender**		
Male	98 (69.0)	72 (32.1)
Female	44 (31.0)	152 (67.9)
Marital Status**		
Married	106 (74.6)	82 (36.6)
Widowed	36 (25.4)	142 (63.4)

*Note*.

* indicates *p* < 0.05;

** indicates *p* < 0.001. Participants who scored above the threshold in at least one psychiatric disorder screening tool were counted as a “positive screen.” Participants who did not score above the threshold were counted as “normal.”

**Table 3:** Participants who screened positive for psychiatric disorders stratified by age group

	Age Group *N* (%)					

	60 – 64	65 – 69	70 – 74	75 – 79	80+	Total
GHQ-12	32	31	35	4	22	124
MMSE	15	7	5	15	18	60
GDS	27	15	11	13	14	80
GA	18	7	4	3	7	39

*Note*. GHQ-12 is the General Health Questionnaire-12. MMSE is the Mini-Mental Status Examination. GDS is the Geriatric Depression Scale. GA is the Generalized Anxiety Scale.

## References

[1] Morley JE (2008). Successful aging or aging successfully. JAMDA.

[2] Palacios R (2002). The future of global ageing. Int J Epidemiol.

[3] Cassel CK (2001). Successful aging. How increased life expectancy and medical advances are changing geriatric care. Geriatrics.

[4] Population Reference Bureau (2012). Today’s research on aging: Issue archive. http://www.prb.org/About/ProgramsProjects/Aging/TodaysResearchAging/IssueArchive.aspx.

[5] Madhu T, Sreedevi A (2013). A study of socio demographic profile of geriatric population in the field practice area of Kurnool Medical College. IJRDH.

[6] Nair SS, Hiremath SG, Ramesh, Pooja, Nair SS (2013). Depression among geriatrics: Prevalence and associated factors. IJCRR.

[7] World Health Organization (WHO) (2012). Depression.

[8] Motohashi Y, Kaneko Y, Sasaki H, Yamaji M (2007). A decrease in suicide rates in Japanese rural towns after community-based intervention by the health promotion approach. Community Ment Health J.

[9] Walston J, Hadley EC, Ferrucci L (2006). Research agenda for frailty in older adults: toward a better understanding of physiology and etiology: summary from the American Geriatrics Society/National Institute on Aging Research Conference on Frailty in Older Adults. J Am Geriatr Soc.

[10] Houttekier D, Cohen J, Bilsen J, Addington-Hall J, Onwuteaka-Philipsen BD, Deliens L (2010). Place of death of older persons with dementia. A study in five European countries. J Am Geriatr Soc.

[11] Mills TL, Cody-Rydzewski S, Chang EC, Downey CA (2012). Psychology of older adults: Exploring the effects of class and culture on the mental health of African Americans. Handbook of race and development in mental health.

[12] Lawson R, Davies BP, Bebbington A, Jamieson A (1991). The UK home help service in England and Wales. Home Care for Older People in Europe: A Comparison of Policies and Practices.

[13] Banerjee S, Macdonald A (1996). Mental disorder in an elderly home care population: Associations with health and social service use. Br J Psychiatry.

[14] Pathak JD (1978). Our elderly: Some effects of aging in Indian subjects.

[15] Nair TK Older people in rural Tamil Nadu.

[16] Venkoba Rao A (1996). National task force study on problems of the aged seeking psychiatric help. I C M R. 1987. Mental health status of the elderly.

[17] Ingle GK, Nath A (2008). Geriatric health in India: Concerns and soluations. Indian J Community Med.

[18] World Health Organization (WHO) (2014). Definition of an older or elderly person. Health statistics and information systems.

[19] Tiwari SC, Srivastava S (1998). Geropsyciatric morbidity in rural Uttar Pradesh. Indian J Psychiatry.

[20] Goldberg DP (1988). A user’s guide to the General Health Questionnaire.

[21] Burns A, Lawlor B, Craig S (2002). Rating scales in old age psychiatry. Br J Psychiatry.

[22] F FM, Folstein SE, McHugh PR (1975). « Mini-mental state ». A practical method for grading the cognitive state of patients for the clinician. J Psychiatr Res.

[23] Yesavage JA, Brink TL, Rose TL (1982–1983). Development and validation of a geriatric depression screening scale: A preliminary report. J Psychiatr Res.

[24] Blacker D, Sadock BJ, Sadock V (2005). Psychiatric rating scales. Comprehensive textbook of psychiatry.

[25] Ramachandran V, Menon SM, Ramamurti P (1979). Psychiatric disorders in subjects aged over fifty. Indian J Psychiatry.

[26] Copeland JR, Dewey ME, Griffiths-Jones HM (1986). A computerized psychiatric diagnostic system and case nomenclature for elderly subjects: GMS and AGECAT. Psychol Med.

[27] Patel V, Kirkwood BR, Pednekar S (2006). Gender disadvantage and reproductive health risk factors for common mental disorders in women: A community survey in India. Arch Gen Psychiatry.

[28] Sood A, Singh P, Gargi PD (2006). Psychiatric morbidity in non-psychiatric geriatric inpatients. Indian J Psychiatry.

[29] Pereira B, Andrew G, Pednekar S, Pai R, Pelto P, Patel V (2007). The explanatory models of depression in low income countries: listening to women in India. J Affect Disord.

[30] Ramsay R, Welch S, Youard E (2001). Needs of women patients with mental illness. BJPsych Advances.

[31] Steadman HJ, Osher FC, Robbins PC, Case B, Samuels S (2009). Prevalence of serious mental illness among jail inmates. Psychiatr Serv.

[32] Lindesay J, Briggs K, Murphy E (1989). The Guy’s/Age Concern survey. Prevalence rates of cognitive impairment, depression and anxiety in an urban elderly community. Br J Psychiatry.

[33] Beekman AT, Copeland JR, Prince MJ (1999). Review of community prevalence of depression in later life. Br J Psychiatry.

[34] Rao AV, Madhavan T (1982). Gerospsychiatric morbidity survey in a semi-urban area near Madurai. Indian J Psychiatry.

[35] Ritchie K, Artero S, Beluche I (2004). Prevalence of DSM-IV psychiatric disorder in the French elderly population. Br J Psychiatry.

[36] Lorig KR, Ritter P, Stewart AL (2001). Chronic disease self-management program: 2-year health status and health care utilization outcomes. Med Care.

[37] Gill TM, Desai MM, Gahbauer EA, Holford TR, Williams CS (2001). Restricted activity among community-living older persons: Incidence, precipitants, and health care utilization. Ann Intern Med.

